# Group B Streptococcus Persistently Colonizing the Adenosquamous Carcinoma of the Lung: A Blessing in Disguise?

**DOI:** 10.7759/cureus.57377

**Published:** 2024-04-01

**Authors:** Muhammad Humayoun Rashid, Tehmina Habib, Syeda Neelam Yamin Bukhari, Faiqa Riaz, Diana Kolman-Taddeo

**Affiliations:** 1 Internal Medicine, Capital Health Regional Medical Center, Trenton, USA; 2 Internal Medicine, Nishtar Medical University, Multan, PAK; 3 Internal Medicine, Sir Syed Medical College, Karachi, PAK; 4 Interventional Pulmonology, Capital Health Regional Medical Center, Trenton, USA

**Keywords:** streptococcus agalactiae, lung cancer, pneumonia, squamous cell carcinoma (scc), group b streptococcus (gbs)

## Abstract

*Group B Streptococcus* (GBS), also known as *Streptococcus agalactiae*, is a gram-negative, beta-hemolytic facultative anaerobe that causes neonatal pneumonia and sepsis. The neoplastic epithelial cells in adults, especially those of squamous origin, can show special adhesive properties toward GBS, which tends to reside within these tumors. There are some animal and human studies proving this association. Here, we present a 64-year-old female patient who had lung carcinoma of mixed adeno and squamous origin found to have persistent GBS every time the bronchoscopy was done for tumor ablation or cryotherapy. Subsequently, after starting her on chemo-radiotherapy, she also presented with multiple episodes of pneumonia caused by GBS and *Pseudomonas aeruginosa.* Moreover, many animal studies have shown the anti-tumor properties of GBS toxin that can prevent its metastasis and stop vascular growth surrounding the tumor. This property of GBS toxin can prove a blessing in disguise.

## Introduction

*Group B Streptococcus*(GBS), also known as *Streptococcus agalactiae*, is a gram-positive, beta-hemolytic, catalase-negative, and facultative anaerobe [1]. Generally, GBS is a commensal bacterium that lives inside the human body, colonizing the gastrointestinal and lower genital tract, and 30% of the population acts as asymptomatic carriers [2]. However, in some populations, it can also cause invasive diseases, especially in neonates, the elderly, and immunocompromised people. The virulence factors produced by GBS include polysaccharide capsules and beta hemolysin toxin. Most common diseases caused by GBS include sepsis, urinary tract infections, pneumonia, and endocarditis [3] Studies are present that have demonstrated the attachment of GBS to host epithelial cells via different adhesion receptors, including fibronectin, lamination, and cytokeratin [4]. One of the studies specifically demonstrated that GBS can attach to two forms of cytokeratin 8 that are present particularly in the pulmonary epithelial cells of neonates or the pulmonary neoplastic cells of some adults suffering from lung carcinoma [5]. This adherence can generate inflammatory responses, which can cause disease in neonates and stop the metastatic proliferation of cancer cells. Here, we encountered a case of a female suffering from mixed adeno plus squamous-type lung carcinoma that was causing bronchial obstruction and lung collapse, which had persistent group B streptococcal colonization.

## Case presentation

A 64-year-old female with a past medical history of hypertension, hyperlipidemia, chronic sinusitis, and atrial fibrillation came into the hospital with shortness of breath. She had a smoking history of one pack/day for 30 years. She had a CT calcium scoring done that showed incidental left lower lobe mass with adenopathy and atelectasis. Subsequent CT of the chest showed a subcarinal mass-like process with adenopathy and an infra-hilar mass lesion with occlusion of the right lower lobe bronchus with tumor involvement around the left main stem bronchus. The patient underwent bronchoscopy with a biopsy showing a polypoid tumor in the left lower lobe with complete obstruction.

**Figure 1:**
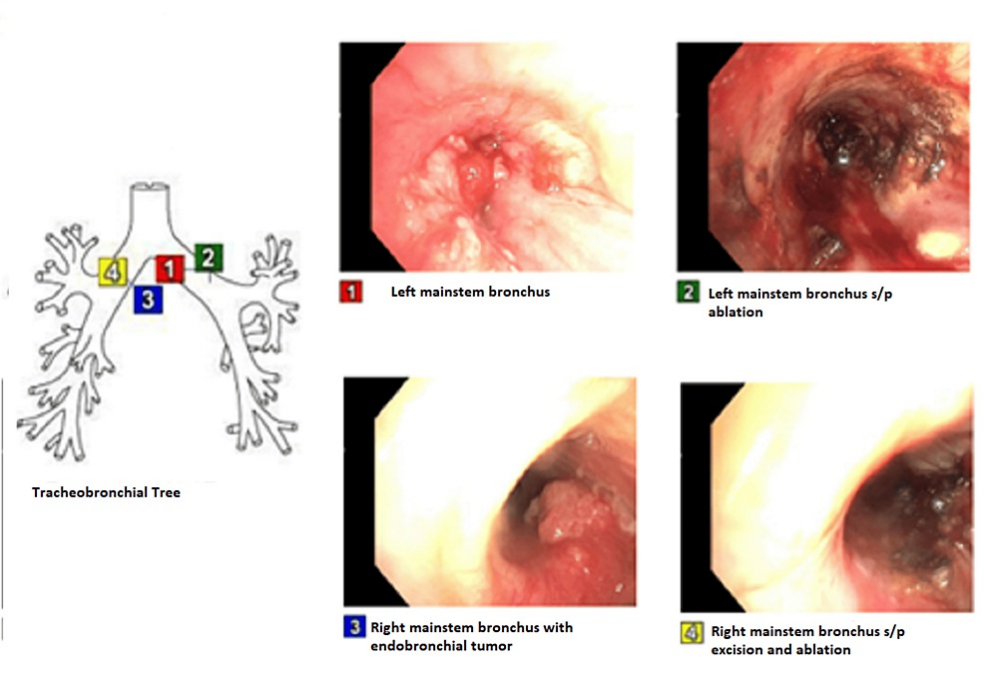
Bronchoscopy showing endobronchial tumor both pre- and post-excision/ablation

Biopsy showed non-small cell adeno and squamous mixed carcinoma. Subsequently, she started having increased dyspnea, and a repeat bronchoscopy one month later showed complete obstruction of the left upper and lower lobes with tumor extending and narrowing the left main stem. A total of 40% of the right main stem obstruction with endobronchial tumor was present. The tumor was biopsied and excised, followed by ablation using electrocautery, argon plasma coagulation (APC), and cryotherapy, as shown in Figure 1.

**Figure 2:**
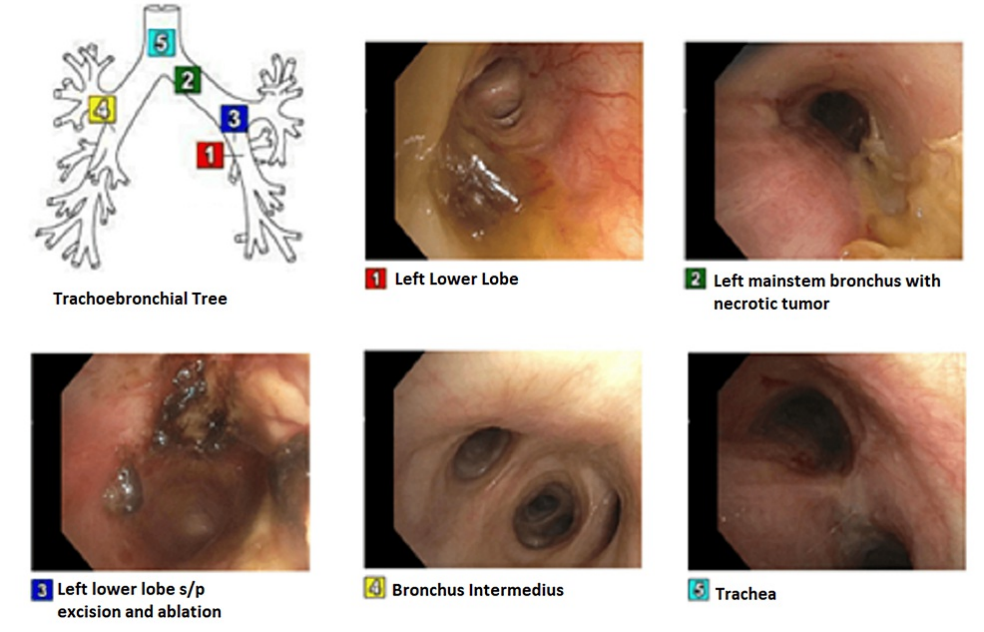
Second bronchoscopy with repeat excision and ablation

Bronchoalveolar lavage at that time showed GBS colonization but the patient had no signs of pneumonia. She was started on combined external beam radiotherapy with high-dose-rate brachytherapy for the left lung for reopening her airway with a follow-up PET-CT scan for total staging and further treatment recommendations. At that time, there was no evidence of distant metastasis. Another bronchoscopy was performed two months later with necrotic mass removal and ablation, as shown in Figure 2, and bronchoalveolar lavage was done, which did not grow any bacteria.

**Figure 3:**
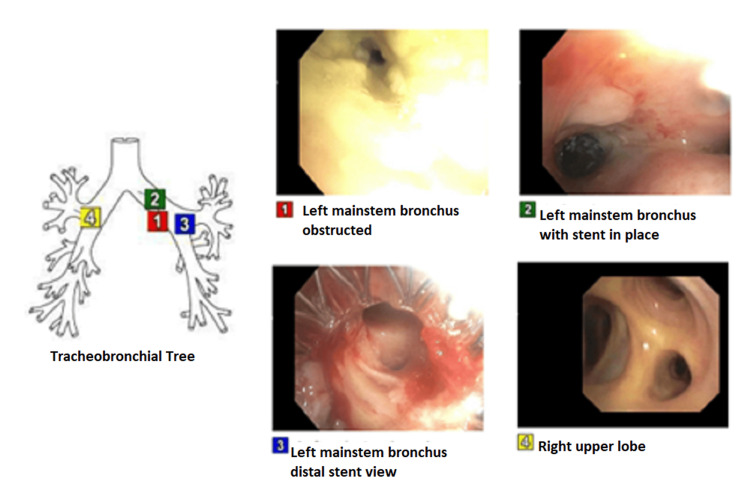
Third bronchoscopy with repeat tumor debulking/ablation and endobronchial stent placement

The patient then presented to the hospital with acute hypoxemic respiratory failure secondary to pneumonia. A repeat bronchoscopy was done and she was found to have *group B streptococcal* pneumonia and was treated with IV cefazolin with repeat bronchial washing culture showing elimination of bacteria after antibiotic therapy. She was then discharged from the hospital to continue her outpatient lung cancer treatment. Subsequently, after four months, the patient presented again with acute hypoxemic respiratory failure with productive cough and yellow sputum, found to have left basilar consolidation and left upper lobe infiltrates. CT scan of the chest showed worsening in the narrowing of the left main stem bronchus and repeat bronchoscopy with tumor debulking/ablation and endobronchial stent placement was done, as shown in Figure 3.

Bronchoalveolar lavage at that time again showed GBS along with *Pseudomonas aeruginosa*. The patient was treated with appropriate antibiotics, which resulted in the resolution of pneumonia, and she was discharged again. She also developed metastasis of lung carcinoma to the liver. The patient had two other subsequent admissions for pneumonia caused by *Pseudomonas aeruginosa* and GBS. Unfortunately, the patient died of cardiopulmonary arrest.

## Discussion

The patient discussed above was diagnosed with mixed-type adeno and squamous carcinoma that was obstructing the bronchus of the left lung. The patient presented with multiple episodes of pneumonia and each time the bronchoalveolar lavage showed group B streptococci colonization and the patient improved after antibiotic treatment. GBS pneumonia is otherwise rare to find in adults and is mostly associated with neonatal pneumonia and sepsis. This raises the concern that the tumor cells have some kind of adhesive properties for the group B streptococci that make it easier to colonize and difficult to eliminate.

Adherence of GBS to the respiratory epithelial cells is considered to be the first step in its virulence pathway. Various components are present on the surface of GBS, including capsular polysaccharides, surface proteins, lipoteichoic acid, and group antigens. Different studies have demonstrated that adherence of GBS to epithelial cells involves surface proteins [6]. In addition to fibronectin and laminin, cytokeratin is also involved in the adherence of GBS to epithelial cells. Different types of cytokeratin are present within the epithelial cells; some are present on the surface of epithelial cells while others are expressed at the cytoplasmic side of the plasma membrane. A study was conducted to prove the adherence of GBS to a specific type of cytokeratin known as cytokeratin 8 (CK8) that is present on the cytoplasmic side of the plasma membrane of pulmonary epithelial cells of neonates and also on pulmonary neoplastic cells of adults suffering from lung carcinoma [5]. This study used A549, an epithelial cell lining from human lung carcinoma, which has many characteristics of type I alveolar pneumocytes that were obtained from the American Type Culture Collection. CK8 plays a role in the adherence of plasma membranes to the cytoskeleton. This study demonstrated that GBS does bind to CK8, and this can occur through multiple mechanisms. Firstly, GBS can either invade the epithelial cells by using other virulence factors or use surface proteins to attach to CK8 on the cytoplasmic side of the plasma membrane. Secondly, it may attach directly on the surface of epithelial cells to the CK8 expressed by pulmonary malignant epithelial cells. Thirdly, it can attach to other types of cytokeratin on the surface of epithelial cells that are still not studied [5]. It is also postulated that GBS can secrete hemolysin/cytolysin that can damage the respiratory epithelial layer resulting in the expression of CK8 present on the cytoplasmic side of epithelium to which GBS can adhere. Hembrough et al. also identified CK8 on the surface of hepatocytes and malignant epithelial cells [7].

GBS toxin is a polysaccharide toxin that is produced by GBS bacteria. This toxin is responsible for causing respiratory distress syndrome in neonates. It causes capillary endothelial damage of the neonatal lung parenchyma during the early few days after birth. This ability of pulmonary endothelial cells to bind to GBS toxin is lost after the neonatal period. Therefore, GBS colonization is rare to find in adults. However, the developing endothelium of neoplastic lesions was found to have the adhesive properties of GBS toxin confirmed by immunohistochemistry. Gray B. Thurman and his colleagues did an animal study on the mice bearing transplanted Madison lung tumors and saw the effect of GBS toxin on it [8]. They found that an inflammatory response can be induced by the IV infusions of GBS toxin around the vasculature of neoplastic lesions that can significantly reduce tumor growth. Some tumors regressed in size initially but recurred after stopping the treatment. Another animal study was done on mice bearing human tumor xenograft [9]. Mice were exposed to intravenous GBS toxin, which caused tumor necrosis, hemorrhagic lesions, and change in tumor morphology, recruitment of lymphocytes and macrophages, and capillary thrombosis. During the studies, no damage was observed to the vasculature of healthy tissue. This study shows the potential antitumor effects of GBS toxin. After this, Hellerqvist and colleagues did phase 1 human trials on 15 patients with different malignancies using group B streptococcal toxin (CM101) [10]. They found that CM101 can initiate inflammatory responses in the neo-vasculature of human malignancies in the same way as that of IV infusions of tumor necrosis factor-alpha (TNF-alpha) and other cytokines. This toxin, when administered, resulted in the elevation of inflammatory cytokines, including TNF-alpha, interleukin-10, and interleukin-8, in the first 12 hours of treatment. They also found partial regression of two tumors and a significant reduction in one tumor. The tumor that showed the best response was Mediterranean-type Kaposi sarcoma.

Therefore, when patients with lung carcinoma present with pneumonia,group B streptococcal colonization should be highly suspected and prompt treatment should be provided. However, the antitumor properties of GBS toxin on human neoplastic lesions are still under evaluation, and it is debatable whether GBS toxin can prevent tumor metastasis by inducing an inflammatory response and destroying tumor vasculature. But one thing is certain that GBS has some specific adhesive properties toward neoplastic epithelial cells.

## Conclusions

It is concluded that neoplastic epithelial cells, especially those of squamous origin, have special adhesive properties toward GBS. Therefore, for patients who have lung carcinoma and present with recurrent pneumonia, empiric treatment should also include coverage for GBS. Moreover, many animal studies have shown the anti-tumor properties of GBS toxin that can prevent its metastasis and stop vascular growth surrounding the tumor. This property of GBS toxin can prove a blessing in disguise.

## References

[1] Raabe VN, Shane AL (2019). Group B Streptococcus (Streptococcus agalactiae). Microbiol Spectr.

[2] Rajagopal L (2009). Understanding the regulation of Group B streptococcal virulence factors. Future Microbiol.

[3] Edwards MS, Baker CJ (2005). Group B streptococcal infections in elderly adults. Clin Infect Dis.

[4] Tamura GS, Rubens CE (1995). Group B streptococci adhere to a variant of fibronectin attached to a solid phase. Mol Microbiol.

[5] Tamura GS, Nittayajarn A (2000). Group B streptococci and other gram-positive cocci bind to cytokeratin 8. Infect Immun.

[6] Tamura GS, Kuypers JM, Smith S, Raff H, Rubens CE (1994). Adherence of group B streptococci to cultured epithelial cells: roles of environmental factors and bacterial surface components. Infect Immun.

[7] Hembrough TA, Kralovich KR, Li L, Gonias SL (1996). Cytokeratin 8 released by breast carcinoma cells in vitro binds plasminogen and tissue-type plasminogen activator and promotes plasminogen activation. Biochem J.

[8] Thurman GB, Russel BA, York GE, Wang YF, Page DL, Sundell HW, Hellerqvist CG (1994). Effects of group B Streptococcus toxin on long-term survival of mice bearing transplanted Madison lung tumors. J Cancer Res Clin Oncol.

[9] Harris AL (1997). Clinical trials of anti-vascular agent group B Streptococcus toxin (CM101). Angiogenesis.

[10] Hellerqvist CG, Thurman GB, Page DL, Wang YF, Russell BA, Montgomery CA, Sundell HW (1993). Antitumor effects of GBS toxin: a polysaccharide exotoxin from group B beta-hemolytic Streptococcus. J Cancer Res Clin Oncol.

